# Fcγ receptors on aging neutrophils

**DOI:** 10.1590/1678-7757-2020-0770

**Published:** 2021-03-31

**Authors:** Thaís Helena GASPAROTO, Thalita Marcato DALBONI, Nádia Ghinelli AMÔR, Aneli Eiko ABE, Graziela PERRI, Vanessa Soares LARA, Narciso Almeida VIEIRA, Carlos Teodoro GASPAROTO, Ana Paula CAMPANELLI

**Affiliations:** 1 Universidade de São Paulo Faculdade de Odontologia de Bauru Departamento de Ciências Biológicas BauruSP Brasil Universidade de São Paulo, Faculdade de Odontologia de Bauru, Departamento de Ciências Biológicas, Bauru, SP, Brasil.; 2 Universidade de São Paulo Faculdade de Odontologia de Bauru Departamento de Estomatologia BauruSP Brasil Universidade de São Paulo, Faculdade de Odontologia de Bauru, Departamento de Estomatologia (Patologia Oral), Bauru, SP, Brasil.; 3 Hospital de Anomalias Craniofaciais BauruSP Brasil Hospital de Anomalias Craniofaciais (HRAC), Bauru, SP, Brasil.; 4 Universidade de São Paulo Faculdade de Medicina de São Paulo Departamento de Saúde Pública São Paulo Brasil Universidade de São Paulo, Faculdade de Medicina de São Paulo, Departamento de Saúde Pública, São Paulo, Brasil.

**Keywords:** Receptors, IgG, Neutrophils, Aging

## Abstract

**Objective:**

Neutrophils are key effector cells of the innate immune system. They recognize antigens through membrane receptors, which are expressed during their maturation and activation. Neutrophils express FcγRII (CD32), FcγRIII (CD16), and FcγRI (CD64) after being activated by different factors such as cytokines and bacterial products. These receptors are involved with phagocytosis of IgG-opsonized microbes and enhance defense mechanisms. Based on that, our study seeks to compare the expression of FcγRII, FcγRIII, FcγRI, and CD11b on neutrophils from elderly and young subjects and their expression after *in vitro* activation with cytokines and LPS.

**Methodology:**

Neutrophils were isolated from human peripheral blood and from mice bone marrow by density gradient. After isolation, FCγRs expression was immediately analyzed by flow cytometry or after *in vitro* stimulation.

**Results:**

In freshly isolated cells, the percentage of FcγRIIIb^+^ and CD11b^+^ neutrophils were higher in samples from young individuals; FcγRIIIa expression was more prominent on aged neutrophils; FcγRIA expression was similar in all samples analyzed. Exposure to CXCL8 and LPS resulted in a higher percentage of FcγRIa^+^ neutrophils on elderly individuals’ samples but lower when compared with neutrophils from young donors. We observed that LPS caused an increase in FcγRIIa expression on aging human neutrophils. In contrast, FcγRIIIb expression in response to CXCL8 and LPS stimulation was not altered in the four groups. CD11b expression was lower in neutrophils from elderly individuals even in response to LPS and CXCL8. In mice, we observed differences only regarding CD11b expression, which was increased on aged neutrophils. LPS exposure caused an increase in all FcγRs.

**Conclusions:**

Our results suggest that, in humans, the overall pattern of FcγR expression and integrin CD11b are altered during aging and immunosenescence might contribute to age-related infection.

## Introduction

Neutrophils are the first inflammatory cell to infiltrate inflamed or infected tissue; therefore, they are recognized as major components of the host’s first line of defense.^1^ They immediately migrate to the infected site, become activated, and initiate a cascade of defense mechanisms that effectively contribute to the development of the adaptive immune response.^2,3^ Several cytokines, such as CXCL8 and TNF, have been described as chemoattractants and activators for neutrophils.^3,4^ Basal levels of CXCL8, the most neutrophil attractant and activator, are detected in the peripheral blood of healthy subjects, but certain diseases can cause CXCL8 deregulation and affect neutrophil function.^5,6^

Neutrophils motility and activity rely on the expression of specialized membrane molecules, which allows them to adhere and transmigrate through the endothelium as well as to recognize and phagocytize opsonized microorganisms.^1^ Among them, the family of Fc gamma receptors for IgG (FcγRs) plays an essential role during neutrophils activation and function. FcγRs mediate antibody-dependent cellular cytotoxicity (ADCC) and the phagocytosis of opsonized invading pathogens, and elicit neutrophil recruitment.^3,7^ The most well-studied FcγRs are the ones that belong to the family of type I FcγRs.^8^ Specifically, the FcγRIIIb (CD16) is the most abundant FcγR;^9^the FcγRIIa (CD32) is an activating-type Fc receptor and is the second most expressed FcγR;^10^ and the less abundant FcγR is the high-affinity receptor FcγRIa (CD64), which binds to IgG2a (mice) or IgG1 and IgG3 (humans).^9^

Since the humoral immune response is essential for host immunity, FcγRs expression has been extensively investigated in diseases and therapies.^10,11^ However, the mechanisms of how immunosenescence affect these receptors’ expression and function, especially on neutrophils, remains poorly investigated. It is well recognized that the aging process hampers the host’s ability to develop robust and effective immune response.^12,13^ Studies have identified differential expression of several receptors on neutrophils from elderly adults as well as distinct activation patterns that were associated with *Candida*-related denture stomatitis.^14,15^ Since alterations in FcγRs and CD11b expression might contribute to immunosenescence and age-related diseases, we aimed at investigating if age affects FcγRII, FcγRIII, FcγRI, and CD11b expression on human and murine neutrophils.

## Methodology

### Subjects

Thirty healthy subjects from two age groups were recruited in Bauru, state of São Paulo, Brazil, and enrolled in our study: elderly group (62 - 75 years; mean ± SD 66.9±4.2; n=15, 08 women and 07 men); and young group (20 - 49 years; mean ± SD 26.4±10.6; n=15, 07 women and 08 men). The age range was determined based on a previous study.^16^ Exclusion criteria for all subjects included the existence of any systemic infectious disease, cancer, endocrine, immune or hematologic alterations and tobacco consumption. We also dismissed volunteers that were under chemo- or radiotherapy, under antibiotics or antifungal medications; or psychotic, heavy metal, anticonvulsant, or cardiotonic treatment. The study was approved by the local Research Ethics Committee of Bauru School of Dentistry (CAAE 41907015.2.0000.5417) and written informed consent was obtained from each patient.

### Animals

Procedures were conducted in accordance with the Guidelines for the Care and Use of Laboratory Animals (CONCEA) and approved by the Institutional Animal Care and Use Committee of the Bauru School of Dentistry, University of São Paulo (Protocol #0042/2013). In total, 6-8-week-old female and male mice aged (young) and 8-month-old (middle-aged) C57BL/6 were provided by the School of Medicine of Ribeirão Preto, University of São Paulo. Each mouse was housed in an isolated cage, and food and water were provided *ad libitum*.

### Isolation and culture of human neutrophils

Human neutrophils were purified from the peripheral blood of healthy donors immediately after collection by density gradient centrifugation using the Histopaque 1119 and 1083 gradients (Sigma-Aldrich) as previously described.^17^ Neutrophils were identified by staining with Turk’s solution and positivity for FITC-conjugated anti-human CD66b (clone G10F5) was established by flow cytometry. Neutrophil stimulation was performed with 100 ng/mL LPS from *Escherichia coli* (Sigma) and/or 10 ng/mL CXCL8 (BD Biosciences Corp., San Diego, CA).

### Cytokine assay

CXCL8 levels were measured using ELISA kits (BD Biosciences), according to the manufacturer’s instructions

### Isolation of murine neutrophils

Murine neutrophils were purified from the bone marrow. Femurs and tibiae of C57Bl/6 mice were dissected, decontaminated with 70% ethanol for 2 min, and then washed in PBS. Then, the bone marrow was flushed with RPMI 1640 medium (Gibco, Grand Island, NY), and layered on the Histopaque 1017 and 1119 gradients (Sigma-Aldrich) and centrifuged as previously described.^18^ Neutrophil stimulation was performed with 100 ng/mL LPS from *Escherichia coli* and/or 10 ng/mL TNF (Sigma-Aldrich).

### FACS analysis of neutrophils

FACS assays were also used to measure Fcγ receptors on neutrophils. For immunostaining of human neutrophils, primary antibodies used were FITC-conjugated anti-human CD66b (clone G10F5), APC-conjugated anti-human CD16 (FcγRIIIb, clone 3G8), PE-conjugated anti-human CD32 (FcγRIIa, 3D3), PE-conjugated anti-human CD64 (FcγRIa, 10.1), and PE-conjugated anti-human CD11b (ICRF44). For immunostaining of mice neutrophils, primary antibodies used were: FITC-conjugated anti-mouse Ly-6G (Gr-1, clone RB6-8C5), FITC-conjugated anti-mouse FcγRIII/FcγRII, Alexa-fluor 647-conjugated anti-mouse FcγRI, and PE-conjugated anti-mouse CD11b were used. Isotype primary antibody controls were used to assess nonspecific staining. Staining was done for 30 minutes at 4°C for all antibodies. All antibodies were obtained from BD Pharmingen. Cell acquisition (counting) was performed on a FACSort flow cytometer using the CellQuest software (BD Biosciences).

### Statistical Analysis

Values are represented as mean ± SD in each figure. Comparisons between two groups were made with a *t*-test followed by the Mann-Whitney test for nonparametric data or with Welch´s correction for parametric data. Comparisons of three groups were made using two-way ANOVA followed by Tukey´s or Sidak’s multiple comparisons test. p≤0.05 were considered as statistically significant.

## Results

Altered frequency of FcγR^+^ and CD11b^+^ on aging human neutrophils

**Figure 1:**
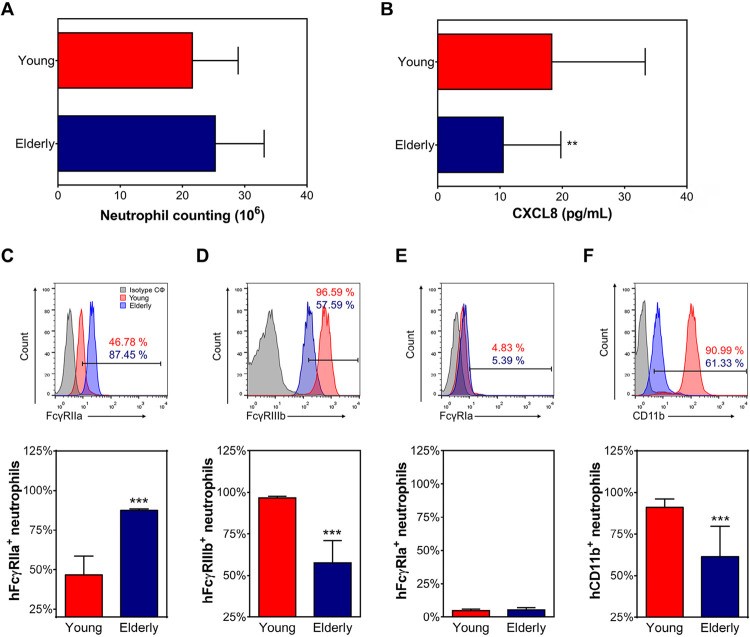
CXCL8 levels and expression of FcγRIIIb and CD11b on neutrophils from healthy elderly individuals are decreased.

We started evaluating the number of neutrophils in human blood. Our results demonstrated that both groups, young and elderly, displayed a similar frequency of neutrophils (Figure 1A). However, we found lower levels of the CXCL8 chemokine, the most important activator for neutrophils,^19^ in samples from elderly subjects when compared with young individuals (Figure 1B). Next, we investigated the FcγRs expression on aging human neutrophils. In freshly isolated human cells, the percentage of FcγRIIa^+^ neutrophils was more evident in samples from elderly individuals (Figure 1C). In contrast, the percentage of FcγRIIIb^+^ neutrophils was significantly higher in the young group (Figure 1D). FcγRIa expression was similar in samples from young and elderly subjects (Figure 1E). Since CD11b is essential for neutrophils extravasation and recruitment;^20^ we also compared its expression on neutrophils from elderly and young individuals and our results showed that the frequency of CD66^+^/CD11b^+^ human neutrophils decreases with age (Figure 1F).

**Figure 2:**
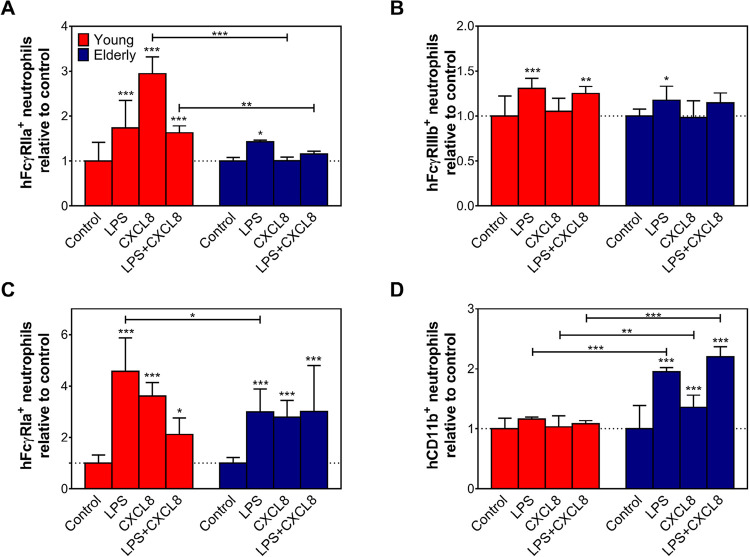
Young human samples present higher FcγR+ neutrophils than older adults’ samples after in vitro activation.

Next, we investigated if LPS or CXCL8 could affect the surface expression of FcγR on neutrophils. We evaluated FcγRIIa, FcγRIIIb, and FcγRIa expression on neutrophils by flow cytometry following 18 hours of exposure with LPS (100 ng/ml), CXCL8 (10 ng/ml), LPS + CXCL8, or medium alone (Figure 2A-C). Exposure to LPS alone significantly increased the expression of all FcγRs receptors in both groups (Figure 2A-C); however, neutrophils from elderly individuals had lower values than the young group, except for FcγRIIIb (Figure 2B). CXCL8 increased the percentage of FcγRIIa^+^ neutrophils in samples from the young group when compared with the elderly group (Figure 2A). Moreover, exposure to CXCL8 increases the frequency of FcγRIa^+^ neutrophils in samples from both groups (Figure 2C). After exposure to CXCL8, no significant difference was observed in the percentage of FcγRIIIb^+^ neutrophils in samples from elderly and young groups (Figure 2B). Culture with LPS plus CXCL8 increased the frequency of FcγRIa^+^ neutrophils in samples from elderly group (Figure 2C). After culture, the percentage of FcγRIIIb^+^ neutrophils in samples from the young group was always lower when compared with *ex vivo* values (data not shown). Despite the lower frequency of CD11b^+^ neutrophils in samples from the elderly group, upon *in vitro* stimulation, its expression was increased. Exposure to LPS or CXCL8 did not increase the percentage of CD11b^+^ neutrophils in samples from young individuals (Figure 2D). Together, the data demonstrate a statistically significant difference in FcγRIIa and FcγRIa expression between the groups, but not between FcγRIIIb expressions in samples from either young or elderly individuals (Figure 2).

The expression of (A) FcγRIIa, (B) FcγRIIIb, (C) FcγRIa, and (D) CD11b on purified neutrophils from young (red bars) and elderly (blue bars) individuals after 18 hours of treatment with LPS, CXCL8, or LPS plus CXCL8 were determined by flow cytometry. Control = neutrophils cultivated without treatment. Data are presented as mean ± SD. The results were evaluated by two-way ANOVA followed by Tukey’s and Sidak’s multiple comparisons test. *p≤0.05, **p≤0.01 and ***p≤0.001.

### Different expression pattern of FcγRIII, FcγRII and FcγRI on activated neutrophils from aged mice compared to young matched controls

**Figure 3:**
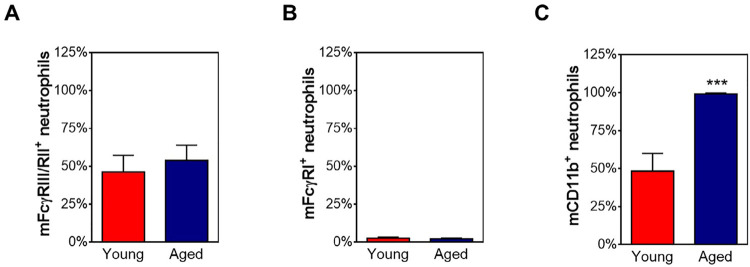
Aged mice have higher frequency of CD11b+ neutrophils.

**Figure 4:**
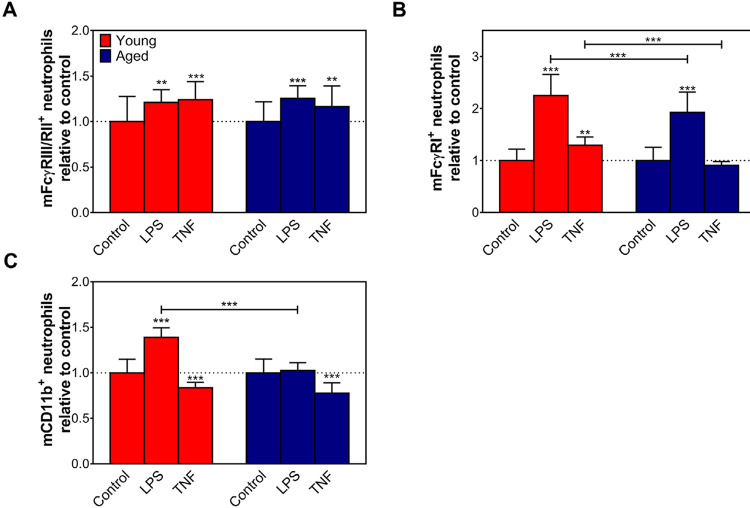
Young murine samples have higher FcγRI+ and CD11+ neutrophils than aged mice after in vitro activation.

FcγRIII, FcγRII, FcγRI, and CD11b expressions were evaluated on bone marrow-derived neutrophils from aged and young mice and analyzed in resting conditions (Figure 3) or after *in vitro* activation with cytokines or LPS (Figure 4). Data obtained clearly showed similar expressions of IgG receptors on neutrophils in a steady state (Figure 3A-B). LPS and TNF promoted increased expression of FcγRIII/FcγRII on neutrophils from young and aged mice (Figure 4A). Moreover, LPS induced a remarkable increase of FcγRI expression on neutrophils from both groups (Figure 4B). However, TNF did not affect this receptor expression by aged cells (Figure 4B). LPS also could stimulate neutrophils from young mice to express CD11b (Figure 4C). Nonetheless, TNF induced a weak decrease in CD11b expression on neutrophils from aged and young mice (Figure 4C).

Representative graphs showing the expression of (A) FcγRIII/FcγRII, (B) FcγRI, and (C) CD11b on purified ex vivo neutrophils from young (red bars) and aged (blue bars) mice determined by flow cytometry. Data are shown as mean±SD. The results were evaluated by t-test. ***p≤0.001.

The expression of (A) FcγRIII/FcγRII, (B) FcγRI, and (C) CD11b on purified neutrophils from young (red bars) and aged (blue bars) mice after 18 hours of treatment with LPS or TNF were determined by flow cytometry. Control=neutrophils cultivated without treatment. Data are shown as mean±SD. The results were evaluated by two-way ANOVA followed by Tukey’s and Sidak’s multiple comparisons test. **p≤0.01 and ***p≤0.001.

## Discussion

The association of age-related disorders with inflammatory pathogenesis^21,22^ shows the great impact of immunosenescence in older adults’ quality of life. Previous work from our laboratory demonstrated that elderly individuals exhibit differences in the phenotype of circulating or tissue-infiltrating neutrophils, associated with oral dysbiosis and development of stomatitis.^14,15,17^ Type I FcγRs play essential roles in immune responses to pathogens and are expressed by several innate immune cells, including neutrophils. However, the impact of immunosenescence in FcγRs remains poorly understood. In our article, we demonstrated that, in humans, aging decreases the frequency of FcγRIIb^+^ neutrophils and could negatively modulate FcγRIIa and FcγRIIIb expression by neutrophils upon stimulation with cytokine and LPS.

The results obtained show decreased levels of CXCL8 in the serum from the older adults when compared with young individuals. Considering that basal levels of CXCL8 are found in the blood serum from healthy individuals, maintaining neutrophils in the homeostasis and, probably, controlling their phenotype,^23^ we speculated if CXCL8, the most neutrophil activator factor, would be at different levels on the blood and affect FcγRs expression on neutrophils. Elderly individuals displayed significantly less FcγRIIIb^+^ neutrophils when compared with young subjects, which is in accordance with a previous study.^24^ Furthermore, CXCL8 was not sufficient to increase FcγRIIIb^+^ neutrophils, suggesting that this chemokine is not important to FcγRIIIb expression on human neutrophils.^25^

Indeed, our results showed that the stimulation with CXCL8 did not induce FcγRIIa expression on neutrophils from elderly subjects. The percentage of the second most abundant FcγRs, the FcγRIIa, was more prominent on freshly isolated neutrophils from elderly individuals, suggesting the existence of a compensatory mechanism of FcγRIIIb/FcγRIIa expression during immunosenescence. We also demonstrated that, although LPS increased FcγRIIa expression on neutrophils from the elderly group, FcγRIIa induction upon other stimulation is lower when compared with neutrophils obtained from young individuals, suggesting that aged neutrophils probably require a more robust signaling activation. Neutrophil activation by FcγR usually requires the co-engagement of FcγRIIIb and FcγRIIa,^26^ and upon efficient cross-linking, neutrophils produce inflammatory mediators and reactive oxygen species, and trigger neutrophil extracellular trap (NET) formation,^7,27^ so the lower frequency of FcγRIIa^+^ neutrophils detected in samples from older adults after *in vitro* stimulation might imply in lower neutrophil activation.^28^ Concerning mice neutrophils, our results showed no significant differences in the expression of FcγRIII/FcγRII in freshly isolated neutrophils from young and aged mice. LPS and TNF induced an increase of FcγRIII/FcγRII^+^ neutrophils in both groups.

We also determined percentages of FcγRIa^+^ neutrophils, the less abundant FcγR, before and after stimuli, and, as expected, both human neutrophils express low FcγRIa regardless of age. Although FcγRIa expression is minimal on resting neutrophils,^29^ it can be upregulated after activation.^30,31^*In vitro*, LPS markedly increased the frequency of FcγRIa^+^ neutrophils. However, the induction of FcγRIa was even higher in young individuals when compared with older individuals. FcγRIa upregulation allows neutrophils to efficiently trigger antibody-dependent cytotoxicity (ADCC).^32^ Thus, as suggested for FcγRIIIb, neutrophils from elderly individuals might need stronger signal receptor assembling and signaling pathways to trigger FcγRIa expression or even other inflammatory-induced receptors.^33^

CD11b^+^ neutrophils were more abundant in the blood from young subjects, where aged mice had a higher frequency of CD11b^+^ neutrophils than young ones. However, the groups that displayed higher numbers of neutrophils expressing CD11b did not increase its expression effectively upon *in vitro* stimulation. Studies evaluating the effects of immunosenescence on CD11b expression are scarce and conflicting. It has been reported that CD11b can be both unaltered and increased with age. CD11b is an integrin that forms complexes with CD18 and regulates adhesion of neutrophils to the endothelium, a fundamental step for leukocyte migration towards inflamed/infected tissue^34^ and its induction is a sign of neutrophil activation.^35^ In addition, CD11b is stored in secretory, gelatinase, and specific granules^36^ and can be rapidly upregulated on stimulated cells by stimulation with bacterial products, including LPS and fMLP.^37^ Since LPS is recognized by different receptors on neutrophils, especially TLR4, which is reduced on neutrophils from older adults; this fact might be influencing LPS-activation of neutrophils.^15^ Although we did not pursue the investigation of the molecular mechanisms underlying the downmodulation of CD11b on human neutrophils, the observation that elderly individuals displayed a lower quantity of systemic CXCL8 might be associated with the decreased frequency of CD11b^+^ neutrophils, since CXCL8 upregulates CD11b expression.^25^ Another possible explanation for the downregulation of CD11b might be related to TNF and CCL3, which decayed prematurely in aged mice during innate granuloma formation^38^ and were demonstrated to induce the expression of CD11b on human neutrophils.^39^ The current demonstration, that elderly subjects had a lower frequency of CD11b^+^ neutrophil, contrasts with an earlier study that reported no significant age-dependent differences in the expression of CD11b.^40^ A possible explication for the difference between studies could be related to differences in cell processing and staining conditions that may result in different levels of surface CD11b and other markers of degranulation.^37^ In our article, we found significant and intriguing differences regarding CD11b expression in elderly humans and aged mice during resting and upon *in vitro* activation, and probably reflects different effects of aging on CD11b expression on neutrophils between both species.

In short, our results suggest that, for neutrophils, immunosenescence results in an imbalance of FcγR and CD11b expression. Moreover, regarding FcγRs and CD11b expression, human and murine neutrophils respond differently to aging and such differences should be considered for the designing of further studies using experimental models for immunosenescence assessment.

(A) Neutrophil counting and (B) CXCL8 levels from peripheral blood from healthy young (red bars) and elderly (blue bars) individuals. Representative histograms and respectively graphs showing the expression of (C) FcγRIIa, (D) FcγRIIIb, (E) FcγRIa, and (F) CD11b on purified ex vivo neutrophils from young (red bars) and elderly (blue bars) individuals determined by flow cytometry. Grey bars are relative to isotype controls. Data are presented as mean±SD. The results were evaluated by t-test. **p≤0.01 and ***p≤0.001.
